# Circulating the HLA-DR+ T Cell Ratio Is a Prognostic Factor for Recurrence of Patients with Hepatocellular Carcinoma after Curative Surgery

**DOI:** 10.1155/2023/1875153

**Published:** 2023-02-23

**Authors:** Rui-Qian Gao, Jing-Han Sun, Yong-Hui Ma, Yu-Wei Xie, Guan-Ming Shao, Cong Wang, Bin Tan, Kui Liu, Kun Li, Na Li, Wei-Yu Hu, Jingyu Cao

**Affiliations:** ^1^Department of Hepatobiliary and Pancreatic Surgery, The Affiliated Hospital of Qingdao University, Qingdao, China; ^2^Department of Cosmetic Surgery, The Affiliated Hospital of Qingdao University, Qingdao, China

## Abstract

**Background:**

HLA-DR+ T cell, accounting for 1.2%–5.8% of peripheral lymphocyte, is a type of activated T lymphocyte. This retrospective study aimed to evaluate the prognostic value of HLA-DR+ T cell for progression-free survival (PFS) and overall survival (OS) in hepatocellular carcinoma (HCC) patients after curative surgery. *Patients and Methods*. Clinicopathological data of 192 patients who underwent curative resection for hepatocellular carcinoma in the affiliated hospital of Qingdao University between January 2013 and December 2021 were collected and analyzed. Statistical tests used in this study were the chi-square test and Fisher's exact test. The prognostic value of the HLA-DR+ T cell ratio was analyzed using univariate and multivariate Cox regression analyses. The Kaplan–Meier curves were drawn by the *R* programming language.

**Results:**

HCC patients were divided into high (≥5.8%) and low (<5.8%) HLADR+ T cell ratio groups. Cox regression analysis indicated that a high HLA-DR+ T cell ratio was positively related to the PFS in HCC patients (*P*=0.003) and AFP-positive (≥20 ng/ml) HCC patients (*P*=0.020). HCC patients and AFP-positive HCC patients in the high HLA-DR+ T cell ratio group were prone to have a higher T cell ratio, a higher CD8+T cell ratio, and a lower B cell ratio than the low HLA-DR+ T cell ratio group. However, the HLA-DR+ T cell ratio was not a statistically significant predictor for OS in HCC patients (*P*=0.57) as well as PFS (*P*=0.088) and OS (*P*=0.63) in AFP-negative HCC patients.

**Conclusions:**

This study confirmed that the HLA-DR+ T cell ratio was a significant predictor of PFS in HCC patients and AFP-positive HCC patients after curative surgery. This association may have guiding significance for the follow-up work of HCC patients after surgery.

## 1. Introduction

Comprising 75%–85% of primary liver cancer, hepatocellular carcinoma (HCC) is one of the most fatal cancers which presents a severe challenge to human health and life quality [1]. Despite recent advances in diagnosis and treatment, the resection rate and overall prognosis of HCC patients are still unsatisfactory. Even if the early-stage HCC patients were treated with curative surgery [2], the incidence of tumor relapse ranges from 50% to 70% [3]. Therefore, it is urgent to identify biomarkers to screen out patients with a high risk of relapse. The tumor immune ecosystem is a key determinant and research hotspot of the progression of HCC [4]. In this study, we analyzed the relationship between HLA-DR+ T cells, one of the peripheral blood lymphocyte subsets, and the prognosis of HCC.

The human leukocyte antigen isotype DR (HLA-DR) as an MHC class II molecule is expressed on antigen-presenting cells (APC) and activated T cells [5, 6]. Numerous detailed studies have elucidated the importance of presenting antigens to T cells by professional APC expressing HLA-DR in the immune process [7, 8]. HLA-DR, as a marker of T cell activation, was shown to associate with disease states, such as cancer, HIV, and autoimmune diseases [9–11]. However, immunosuppressive cells expressing HLA-DR, including TAM tumor-associated macrophage (TAM) and regulatory T (Treg) cells, are involved in immune escape [12, 13]. Additionally, previous studies revealed HCC cells are one major source of HLA-DR in tumor tissues [14]. But in HCC, few studies reported the role of HLA-DR+ T cells in prognosis.

T cells, as the most important part of antitumor immunity, have been the focus of the research. Over these years, more and more studies revealed the significance of HLA-DR+ T cells in the development and treatment of HCC. According to a recent study, mucosal-associated invariant T (MAIT) cells, as innate T cells with immunoregulatory activity, upregulated HLA-DR in peripheral blood of HCC patients but not in hepatic sinusoids and were functionally impaired [15]. The activated but functionally impaired MAIT cells might suggest a potential role in HCC pathogenesis. In addition, the CD57+ HLA-DR+ CD8+ T cells, displaying a highly proliferative and cytotoxic phenotype, were correlated with recurrence in HCC patients after liver transplantation [16]. HLA-DR+ T cells in peripheral are increased in HCC patients with anti-CTLA4 treatment [17]. In conclusion, the role of HLA-DR+ T cells in HCC prognosis deserves further investigation.

We aimed to analyze the relationship between the preoperative HLA-DR+ T cell ratio and postoperative recurrence or death in HCC patients. We also analyzed the differences in baseline data between the groups with different HLA-DR+ T cell ratios to investigate the potential value of HLA-DR+ T cells in HCC progression and prognosis.

## 2. Patients and Methods

### 2.1. Patient Selection

Patients who received liver resection and were pathologically diagnosed as having HCC at the Department of Hepatobiliary and Pancreatic Surgery, The Affiliated Hospital of Qingdao University, between January 2013 and December 2021 were retrospectively collected. We eliminated patients with other malignancies, preoperative anti-HCC treatment, macrovascular invasion, TNM stage III, TNM stage VI, and postoperative liver transplantation for non-neoplastic liver disease, as well as patients lacking complete baseline and follow-up data, resulting in 192 patients included in the study. We acquired clinicopathological data from medical records. This study complied with the ethical guidelines of the Helsinki Declaration, and written informed consent was obtained from participants or their immediate families prior to surgery.

### 2.2. Clinicopathological Variables

The clinicopathological variables of HCC patients incorporated the date of surgery, gender, age, HbsAg, Child-Pugh grade, AFP (alpha-fetoprotein) levels, ALT (alanine transaminase) levels, AST (aspartate transaminase) levels, AST/ALT, GGT (gamma glutamyl transferase) levels, lymphocyte subsets, largest tumor size, tumor number, liver cirrhosis, macro- and microvascular invasion, capsular invasion, tumor satellites, and tumor differentiation. Each subset of lymphocytes was described in terms of its ratio to total lymphocytes. For continuous variables, the upper level of normal (ULN) was used as the cut-off value. AFP positivity was defined as a serum level ≥20 ng/ml. Liver cirrhosis was diagnosed based on pathological findings. The curative surgery referred to complete resection of tumor with negative microscopic margins. The 8^th^ AJCC cancer staging system was used to stage HCC. Partial HCC patients with microvascular invasion received prophylactic TACE (transcatheter arterial chemoembolization) within 1 month after surgery.

### 2.3. Postoperative Follow-Up

All patients were followed up regularly at the outpatient clinic after discharge. AFP and image studies were performed routinely at 1 month after the operation, then at least 3 months in the first 2 years, and at least 6 months thereafter. Dynamic contrast-enhanced computed tomography (CT) of the upper abdomen or Gd-EOB-DPTA-enhanced magnetic resonance imaging (MRI) was performed if digestive ultrasound revealed HCC recurrence and/or alpha fetoprotein (AFP) was significantly elevated. Further auxiliary inspection including chest CT, lower abdominal CT, pelvic CT, or positron emission tomography (PET)-CT was completed when clinically necessary. The diagnosis of tumor recurrence was confirmed by at least two imaging examinations or liver histopathological examination. Once tumor recurrence is found, resurgical resection, local ablation, TACE, radiotherapy, systemic therapy, and supportive care can be selected according to the characteristics of tumor recurrence. The survival information was obtained from clinical follow-up or telephone follow-up. Follow-up was up to September 1, 2021. The starting point of follow-up was the date of surgery; the time interval from the starting point to the date of the first recurrence, first metastasis, death, or last follow-up was PFS; and the time interval from the starting point to the date of death or last follow-up was OS.

### 2.4. Statistical Analysis

Continuous variables were categorized by ULN and described together with categorical variables as frequencies (*N*). The *χ*^2^ test or Fisher's test was performed to compare categorical variables. Using the COX proportional hazard model, we identified independent risk factors associated with postoperative progression or death, with hazard ratios (HR) and 95% confidence intervals (CI). We plotted the Kaplan–Meier curves through the “survival” package of the *R* language software package. SPSS software version 22.0 (SPSS, Chicago, IL, USA) and R language software version 4.1.2 were used for statistical analysis in this study. Results were considered statistically significant when the *P* value was less than 0.05.

## 3. Results

### 3.1. Patient Baseline Information

The baseline clinicopathological characteristics of all HCC patients were outlined in Table 1. A total of 115 (59.9%) patients were less than 60 years old, and 153 (79.7%) patients were male. There were 164 (85.4%) patients who had the history of viral hepatitis B, and 114 (59.4%) patients had liver cirrhosis. There were 187 (97.4%) patients with Child-Pugh grade A, and 5 well selected HCC patients with Child-Pugh grade B who underwent curative surgery were also included in the study according to a comprehensive review [18]. The proportion of patients with AFP positivity (≥20 ng/ml) and a high HLA-DR+ T cell ratio (≥5.8%) were 50.0% and 52.1%, respectively. Except for 2 patients with two tumors, the rest of the patients had one tumor. 190 patients (99.0%) were TNM stage I, and 2 patients were TNM stage II. 116 patients (60.4%) were Edmondson grades I-II. Capsular invasion was noted in 70 (36.5%) patients, and microvascular invasion was noted in 67 (34.9%) patients. After curative surgery, 65 (33.9%) patients received prophylactic TACE. With a median follow-up of 48.3 months in entire study patients, postoperative tumor progression occurred in 81 (42.2%) patients and 32 (16.7%) patients died. Nevertheless, patients with a high HLA-DR+ T cell ratio were less likely to present cancer progression (35%).

### 3.2. The Relationship between Clinicopathological Characteristics and the HLA-DR+ T Cell Ratio

**Figure 1:**
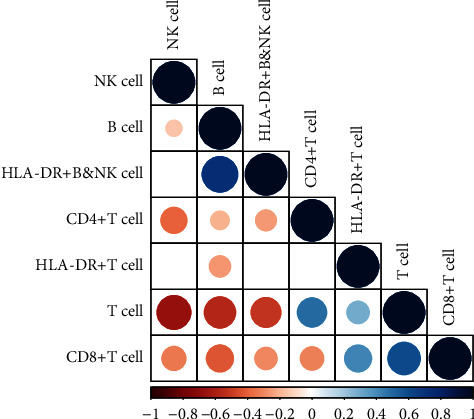
The correlation matrix for 7 lymphocyte ratios. Some lymphocytes were negatively related, represented in orange, and others were positively related, represented in blue. The darker the color, the higher the correlation was (*P* < 0.05).

A total of 100 (52.1%) patients had a high HLA-DR+ T cell ratio. The relationship between clinicopathological characteristics and the HLA-DR+ T cell ratio is shown in Table 1. Patients with a high HLA-DR+ T cell ratio had a higher T cell ratio (*P*=0.005), a higher CD8+ T cell ratio (*P* < 0.001), and a lower B cell ratio (*P*=0.005) than patients with a low HLA-DR+ T cell ratio. In addition, in Figure 1, there is a positive correlation between the HLA-DR+ T cell ratio and the T cell ratio (*P* < 0.05) as well as the CD8+ T cell ratio, while there is a negative correlation between the HLA-DR+ T cell ratio and the B cell ratio (*P* < 0.05).

### 3.3. COX Regression Analyses between Clinicopathological Variables Associated with PFS and OS after Curative Surgery for HCC

**Figure 2:**
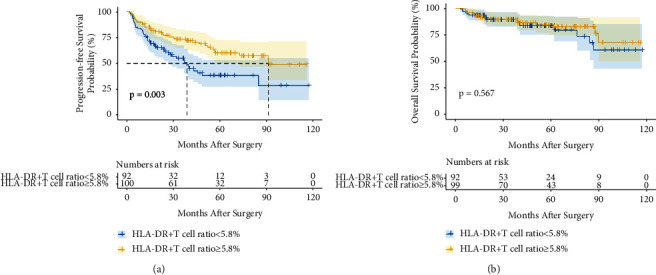
The prognostic value of the HLA-DR+ T cell ratio in patients with HCC. Kaplan–Meier curves of PFS (a) and OS (b) for patients with different HLA-DR+ T cell ratio groups.

As indicated by the Kaplan–Meier curves in Figure 2(a), HCC patients with a high HLA-DR+ T cell ratio had better PFS (*P*=0.003) than those with a low HLA-DR+ T cell ratio; unfortunately, Figure 2(b) shows that the HLA-DR+ T cell was not a prognostic biomarker for OS in HCC patients (*P*=0.567). According to the results of multivariate COX regression analyses, the following features were statistically significant with PFS of HCC patients in Table 2: serum GGT level (*P*=0.027), serum HLA-DR+ T cell ratio (*P*=0.002), tumor Edmondson grade (*P*=0.047), and capsular invasion (*P*=0.045); and the following features were statistically significant with OS of HCC patients in Table 3: serum GGT level (*P*=0.044), largest tumor size (*P*=0.027), and microvascular invasion (*P*=0.030). As a result, the HLA-DR+ T cell ratio was a prognostic factor for PFS but not for OS of HCC patients.

### 3.4. COX Regression Analyses between Clinicopathological Variables Associated with PFS and OS after Curative Surgery for AFP-Positive HCC

**Figure 3:**
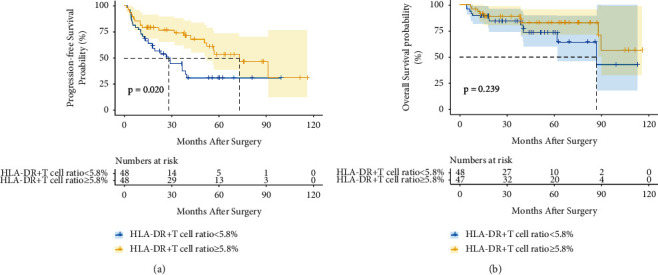
The prognostic value of the HLA-DR+ T cell ratio in AFP-positive HCC patients. Kaplan–Meier curves of PFS (a) and OS (b) for AFP-positive patients with different HLA-DR+ T cell ratio groups.

**Figure 4:**
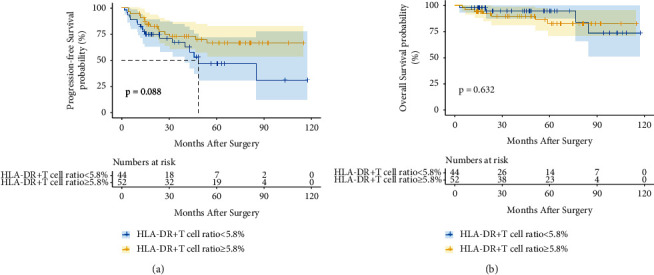
The prognostic value of the HLA-DR+ T cell ratio in AFP-negative HCC patients. Kaplan–Meier curves of PFS (a) and OS (b) for patients with different HLA-DR+ T cell ratio groups.

The clinicopathological characteristics associated with serum AFP positivity (≥20 ng/ml) versus AFP negativity (<20 ng/ml) among HCC patients were noted in Table 4. AFP-positive HCC patients had more female patients (*P*=0.048), higher proportion of HBV infection (*P*=0.041), lower B cell ratio (*P*=0.011), worse tumor Edmondson grade (*P*=0.001), and more microvascular invasion (*P*=0.049) than AFP-negative HCC patients. Figures 3(a) and 3(b) demonstrate that the HLA-DR+ T cell ratio was a prognostic factor for PFS (*P*=0.020) but not for OS (*P*=0.239) in AFP-positive HCC patients. However, Figures 4(a) and 4(b) depict that the HLA-DR+ T cell ratio was nonstatistically significant with PFS (*P*=0.088) and OS (*P*=0.632) of AFP-negative HCC patients. As shown in Tables 5 and 6, COX regression analyses also indicated that the HLA-DR+ T cell ratio was the only prognostic factor for PFS of AFP-positive HCC patients. Similar to the results of all patients in this study, AFP-positive patients with a high HLA-DR+ T cell ratio are more likely to have a high T cell ratio (*P*=0.001), a high CD8+ T cell ratio (*P*=0.020), and a low B cell ratio (*P*=0.022) in Table 7, but AFP-negative HCC patients with a high HLA-DR+ T cell ratio are more likely to have a high CD8+T cell ratio (*P*=0.007) in Table 8.

## 4. Discussion

With the deepening of the research on the immune environment, more and more studies have confirmed that the functional status of T cells plays a pivotal role in the occurrence and development of HCC [4, 19, 20]. As an important molecule for presenting antigens, HLA-DR was mostly used as a target on the surface of APC for HCC-related research [12, 21], whereas HLA-DR+ T cells are shown to be effector T cells in a variety of malignancies and serve as a predictive factor for antitumor treatment [22, 23]. Based on these existing studies, we designed this project to explore the role of HLA-DR+ T cell in the anti-HCC immunity process. To the best of our knowledge, this research first revealed that HLA-DR+ T cell was a predictive marker for recurrence of HCC after hepatectomy.

In this article, we revealed that HCC patients with a high HLA-DR+ T cell ratio were less likely to experience recurrence after curative liver resection. HLA-DR+ cytotoxic T lymphocytes (CTLs) are cytotoxic and express immune signatures of functionally activated cells, such as Granzyme B and IFN-*γ* [24]. Meanwhile, regulatory T (Treg) cells upregulating HLA-DR expression increase in HCC and exhibit enhanced immunosuppressive activity driven by the hypoxia environment [25, 26]. However, it has been recently reported that Treg cells are differently expressed in HCC according to etiology of underlying liver cirrhosis. Treg cells play an immunosuppressive role in chronic viral liver diseases by hindering the antiviral process, while in autoimmune liver diseases, Treg cells show quantitative and functional defects so that they cannot effectively suppress self-reactive lymphocytes [27–29]. Besides, monitoring the genetic background (HLA) of Treg cells has potential value in assessing the extent of drug side effects during treatment with immune checkpoint inhibitors (ICIs) in patients with HCC [29]. HLA-DR is expressed not only on the surface of CTLs but also on immunosuppressive T cells so that the effect of HLA-DR on tumor immunity may be opposite. It is necessary to separately research the expression and prognostic value of HLA-DR in various T lymphocyte subsets. Besides, through the significance test of difference, we demonstrated that patients in the high HLA-DR+ T cell ratio group had a lower B cell ratio than patients in the low HLA-DR+ T cell ratio group. The role of B cells in T cell activation is bidirectional. Tumor-infiltrating B cells activate T cells through spatial cell-to-cell contacts to enhance antitumor activity in HCC patients [30]. On the contrary, B cell-derived GABA (*γ*-aminobutyric acid) impairs cytotoxic T cell responses and antitumor immunity [31]. In this article, we revealed that the B cell ratio was negatively correlated with the activated (HLA-DR+) T cell ratio. In conclusion, we believed that HCC patients with a high HLA-DR+ T cell ratio had a low B cell ratio and were less prone to tumor progression postoperatively.

AFP, as a tumor-associated antigen (TAA) of HCC [32], is considered a serum biomarker for diagnosis and a potential target for immunotherapy [33]. We, respectively, analyzed the prognostic significance of the HLA-DR+ T cell ratio in AFP-positive and AFP-negative HCC patients. There was no statistically significant difference in the HLA-DR+ T cell ratio between AFP-positive and AFP-negative HCC patients. But only among AFP-positive HCC patients, we found that patients in the high HLA-DR+ T cell ratio group had a lower rate of postoperative tumor progression than patients in the low HLA-DR+ T cell ratio group. It has been reported that AFP has strong binding properties towards HLA-DR of helper T lymphocyte and T cell response against AFP contributes to the significantly improving survival rate [34]. As part of the normal T cell repertoire, TAA-specific T cell responses are important for controlling HCC in different stages [35, 36]. However, further research will be needed to confirm whether HLA-DR+ T cells are AFP-specific T cells.

This study aimed to find new predictors of postoperative recurrence to optimize postoperative follow-up protocols. For the first time, HLA-DR+ T cell was used as a marker of antitumor immunity activity in the study of HCC. Simultaneously, we conducted stratification studies and demonstrated the specific prognostic value of HLA-DR+ T cells for postoperative recurrence in AFP-positive HCC patients. Moreover, apart from the HLA-DR+ T cell ratio, this study found other predictors for progression of HCC patients after curative resection, such as the GGT level, Edmondson grade, and capsular invasion.

Meanwhile, there were a number of limitations in the current research. First, this retrospective study in a single center may bring about selection bias. Consequently, a large-scale, multicenter, and prospective study is needed to validate the results of this study. Second, the classification of continuous variables is bounded by the ULN of a single medical institution. Whether the results will differ from those of other hospitals is unknown. Third, this study only included HBV, which is a common etiology of HCC in China, so it cannot be confirmed whether HLA-DR+ T cell have the same prognostic value in HCC caused by other pathogenies. Last, some postoperative adjuvant treatments, such as antiviral therapy, targeted therapy, and immunotherapy, were not included in this study. We need to incorporate these factors affecting postoperative recurrence into the study for further stratified analysis.

## 5. Conclusion

In summary, our study found that the preoperative HLA-DR+ T cell ratio served as a useful prognostic marker for PFS in HCC patients with curative resection, and the same results were obtained in AFP-positive patients. This is beneficial for clinicians to screen patients with poor prognoses based on clinical data in order to improve patient outcomes by developing personalized treatment and follow-up plans.

## Figures and Tables

**Table 1:** Comparisons of clinicopathological characteristics among 192 HCC patients with different HLA-DR+ T cell ratio groups.

Variables		Total (*N*)	High HLADR+ T cell ratio (*N*)	Low HLADR+ T cell ratio (*N*)	*χ* ^2^	*P* value
Age	<60 years	115	55	60	2.082	0.149
	≥60 years	77	45	32		

Gender	Male	153	79	74	0.061	0.805
	Female	39	21	18		

HbsAg	Positive	164	84	80	0.336	0.562
	Negative	28	16	12		

Liver cirrhosis	Yes	114	61	53	0.228	0.633
	No	78	39	39		

AFP level	<20 ng/ml	96	52	44	0.334	0.563
	≥20 ng/ml	96	48	48		

AST/ALT	<1	123	67	56	0.782	0.376
	≥1	69	33	36		

GGT level	<45 U/L	129	70	59	0.749	0.387
	≥45 U/L	63	30	33		

Child-Pugh grade	A	187	97	90		1
	B	5	3	2		

T cell ratio	<77%	150	70	80	8.062	0.005
	≥77%	42	30	12		

CD4+T cell ratio	<41.6%	124	66	58|	0.669
	≥41.6%	68	34	34		

CD8+ T cell ratio	<29.6%	126	54	72	12.052	<0.001
	≥29.6%	66	46	20		

B cell ratio	<18.2%	154	88	66	7.981	0.005
	≥18.2%	38	12	26		

NK cell ratio	<25.6%	168	90	78	1.193	0.275
	≥25.6%	24	10	14		

Largest tumor size	<5 cm	142	71	71	0.948	0.330
	≥5 cm	50	29	21		

Edmondson grade	I-II	116	56	60	1.702	0.192
	III-IV	76	44	32		|
Microvascular invasion	Yes	67	32	35	0.770	0.380
	No	125	68	57		

Capsular invasion	Yes	70	38	32	0.214	0.644
	No	122	62	60		

Prophylactic TACE after surgery	Yes	65	31	34	0.759	0.384
	No	127	69	58		

AFP: alpha-fetoprotein; AST: aspartate aminotransferase; ALT: alanine aminotransferase; GGT: gamma glutamyl transferase; TACE: transcatheter arterial chemoembolization.

**Table 2:** Univariate and multivariate Cox regression analyses of risk factors associated with progression-free survival (PFS) after curative surgery for hepatocellular carcinoma.

Variables	HR comparison	UV HR (95% CI)	UV *P*value	MV HR (95% CI)	MV *P*value
Gender	Male vs. female	1.264 (0.710–2.249)	0.425		
Age	≥60 vs.<60 years	0.774 (0.491–1.219)	0.269		
HbsAg positivity	Yes vs. no	1.499 (0.722–3.113)	0.277		
Liver cirrhosis	Yes vs. no	1.535 (0.957–2.463)	0.076		
AST/ALT	≥1 vs. <1	1.016 (0.634–1.627)	0.948		
GGT level	≥45 U/L vs. <45 U/L	2.104 (1.354–3.271)	0.001	1.692 (1.062–2.696)	0.027
AFP positivity	Yes vs. no	1.648 (1.057–2.568)	0.027	1.371 (0.857–2.194)	0.187
CD3+ T cell ratio	≥77% vs. <77%	0.680 (0.375–1.233)	0.204		
CD4+ T cell ratio	≥41.6% vs. <41.6%	1.129 (0.706–1.803)	0.613		
CD8+ T cell ratio	≥29.6% vs. <29.6%	0.595 (0.363–0.975)	0.040	0.655 (0.387–1.109)	0.116
HLADR+ T cell ratio	≥5.8% vs.<5.8%	0.512 (0.328–0.799)	0.003	0.465 (0.288–0.750)	0.002
B cell ratio	≥18.2% vs. <18.2%	1.427 (0.861–2.366)	0.168		
NK cell ratio	≥25.6% vs. <25.6%	1.063 (0.575–1.956)	0.845		
Edmondson grade	III-IV vs. I-II	1.697 (1.095–2.629)	0.018	1.638 (1.006–2.668)	0.047
Largest tumor size	≥5 vs. <5 cm	1.675 (1.044–2.690)	0.033	1.572 (0.930–2.657)	0.091
Microvascular invasion	Yes vs. No	1.440 (0.924–2.246)	0.108		
Capsular invasion	Yes vs. No	1.709 (1.103–2.647)	0.016	1.615 (1.010–2.582)	0.045
Prophylactic TACE after surgery	Yes vs. No	1.790 (1.154–2.776)	0.009	1.044 (0.633–1.723)	0.867

AFP: alpha-fetoprotein; AST: aspartate aminotransferase; ALT: alanine aminotransferase; GGT: gamma glutamyl transferase; TACE: transcatheter arterial chemoembolization.

**Table 3:** Univariate and multivariate Cox regression analyses of risk factors associated with overall survival (OS) after curative surgery for hepatocellular carcinoma.

Variables	HR comparison	UV HR (95% CI)	UV *P*value	MV HR (95% CI)	MV *P*value
Gender	Male vs. female	1.895 (0.666–5.394)	0.231		
Age	≥60 vs.<60 years	0.594 (0.282–1.251)	0.170		
HbsAg positivity	Yes vs. no	2.223 (0.531–9.302)	0.274		
Liver cirrhosis	Yes vs. no	1.836 (0.823–4.096)	0.138		
AST/ALT	≥1 vs. <1	1.232 (0.588–2.580)	0.581		
GGT level	≥45 U/L vs. <45 U/L	2.459 (1.239–4.880)	0.010	2.156 (1.020–4.558)	0.044
AFP positivity	Yes vs. no	2.241 (1.085–4.626)	0.029	1.534 (0.716–3.287)	0.271
CD3+ T cell ratio	≥77% vs. <77%	0.996 (0.407–2.435)	0.993		
CD4+ T cell ratio	≥41.6% vs. <41.6%	0.412 (0.158–1.072)	0.069		
CD8+ T cell ratio	≥29.6% vs. <29.6%	1.018 (0.500–2.074)	0.960		
HLADR+ T cell ratio	≥5.8% vs.<5.8%	0.861 (0.434–1.707)	0.567		
B cell ratio	≥18.2% vs. <18.2%	1.543 (0.733–3.249)	0.254		
NK cell ratio	≥25.6% vs. <25.6%	1.326 (0.546–3.217)	0.533		
Edmondson grade	III-IV vs. I-II	2.541 (1.269–5.089)	0.008	1.582 (0.727–3.441)	0.247
Largest tumor size	≥5 cm vs. <5 cm	2.885 (1.440–5.780)	0.003	2.346 (1.100–5.007)	0.027
Microvascular invasion	Yes vs. no	2.817 (1.409–5.634)	0.003	2.379 (1.085–5.215)	0.030
Capsular invasion	Yes vs. no	1.757 (0.878–3.515)	0.111		
Prophylactic TACE after surgery	Yes vs. no	2.117 (1.068–4.194)	0.032	0.794 (0.345–1.831)	0.589

AFP: alpha-fetoprotein; AST: aspartate aminotransferase; ALT: alanine aminotransferase; GGT: gamma glutamyl transferase; TACE: transcatheter arterial chemoembolization.

**Table 4:** Comparisons of clinicopathological characteristics among 192 HCC patients with different AFP level groups.

Variables		AFP positivity (*N*)	AFP negativity (*N*)	*χ* ^2^	*P* value
Age	<60 years	62	53	1.756	0.185
	≥60 years	34	43		

Gender	Male	71	82	3.893	0.048
	Female	25	14		

HbsAg	Positive	87	77	4.181	0.041
	Negative	9	19		

Liver cirrhosis	Yes	60	54	0.777	0.378
	No	36	42		

AST/ALT	<1	58	65	1.109	0.292
	≥1	38	31		

GGT level	<45 U/L	61	68	0.369	0.543
	≥45 U/L	35	28		

Child-Pugh class	A	93	94		1
	B	3	2		

T cell ratio	<77%	75	75	0.000	1
	≥77%	21	21		

CD4+ T cell ratio	<41.6%	60	64	0.364	0.546
	≥41.6%	36	32		

CD8+ T cell ratio	<29.6%	61	65	0.369	0.543
	≥29.6%	35	31		

HLADR+ T cell ratio	<5.8%	48	44	0.334	0.563
	≥5.8%	48	52		

B cell ratio	<18.2%	70	84	6.431	0.011
	≥18.2%	26	12		

NK cell ratio	<25.6%	85	83	0.190	0.663
	≥25.6%	11	13		

Largest tumor size	<5 cm	70	72	0.108	0.742
	≥5 cm	26	24		

Edmondson grade	I-II	47	69	10.541	0.001
	III-IV	49	27		

Microvascular invasion	Yes	40	27	3.874	0.049
	No	56	69		

Capsular invasion	Yes	37	33	0.360	0.549
	No	59	63		

Prophylactic TACE after surgery	Yes	36	29	1.140	0.286
	No	60	67		

AFP: alpha-fetoprotein; AFP positivity: AFP ≥ 20 ng/ml; AFP negativity: AFP < 20 ng/ml; AST: aspartate aminotransferase; ALT: alanine aminotransferase; GGT: gamma glutamyl transferase; TACE: transcatheter arterial chemoembolization.

**Table 5:** Univariate Cox regression analyses of risk factors associated with progression-free survival and overall survival after curative surgery for AFP-positive (≥20 ng/ml) hepatocellular carcinoma.

Variables	HR comparison	Progression-free survival		Overall survival	
		UV HR (95% CI)	UV *P* value	UV HR (95% CI)	UV *P* value
Gender	Male vs. female	0.927 (0.479–1.793)	0.822	2.967 (0.684–12.878)	0.146
Age	≥60 vs.<60 years	0.823 (0.449–1.508)	0.529	0.775 (0.311–1.932)	0.585
HbsAg positivity	Yes vs. no	0.770 (0.302–1.962)	0.584	0.896 (0.208–3.859)	0.882
Liver cirrhosis	Yes vs. no	1.386 (0.743–2.587)	0.305	1.180 (0.450–3.096)	0.737
AST/ALT	≥1 vs. <1	0.980 (0.538–1.787)	0.948	1.029 (0.403–2.622)	0.953
GGT level	≥45 U/L vs. <45 U/L	2.122 (1.203–3.745)	0.009	1.974 (0.837–4.656)	0.120
CD3+ T cell ratio	≥77% vs. <77%	0.616 (0.275–1.380)	0.239	0.686 (0.201–2.345)	0.548
CD4+ T cell ratio	≥41.6% vs. <41.6%	1.215 (0.664–2.223)	0.528	0.200 (0.047–0.863)	0.031
CD8+ T cell ratio	≥29.6% vs. <29.6%	0.505 (0.261–0.975)	0.042	0.780 (0.310–1.964)	0.598
HLADR+ T cell ratio	≥5.8% vs. <5.8%	0.499 (0.278–0.895)	0.020	0.589 (0.244–1.421)	0.239
B cell ratio	≥18.2% vs. <18.2%	1.060 (0.569–1.977)	0.854	1.338 (0.551–3.249)	0.520
NK cell ratio	≥25.6% vs, <25.6%	1.680 (0.783–3.603)	0.183	2.816 (1.003–7.909)	0.049
Edmondson grade	III-IV vs. I-II	1.630 (0.908–2.926)	0.101	1.549 (0.648–3.702)	0.325
Largest tumor size	≥5 vs. <5 cm	1.467 (0.796–2.703)	0.219	2.438 (1.021–5.825)	0.045
Microvascular invasion	Yes vs. no	1.538 (0.868–2.729)	0.140	3.514 (1.404–8.797)	0.007
Capsular invasion	Yes vs. no	1.571 (0.888–2.778)	0.121	1.517 (0.625–3.685)	0.357

AFP: alpha-fetoprotein; AST: aspartate aminotransferase; ALT: alanine aminotransferase; GGT: gamma glutamyl transferase.

**Table 6:** Univariate Cox regression analyses of risk factors associated with progression-free survival and overall survival after curative surgery for AFP-negative (<20 ng/ml) hepatocellular carcinoma.

Variables	HR comparison	Progression-free survival		Overall survival	
		UV HR (95% CI)	UV *P* value	UV HR (95% CI)	UV *P* value
Gender	Male vs. female	3.477 (0.828–14.578)	0.088	1.856 (0.237–14.538)	0.556
Age	≥60 vs. <60 years	0.758 (0.377–1.524)	0.436	0.451 (0.119–1.707)	0.241
Liver cirrhosis	Yes vs. no	1.627 (0.786–3.367)	0.190	3.203 (0.689–14.893)	0.138
AST/ALT	≥1 vs. <1	0.958 (0.443–2.072)	0.914	1.635 (0.471–5.678)	0.439
GGT level	≥45 U/L vs. <45 U/L	1.910 (0.938–3.887)	0.074	3.591 (1.083–11.907)	0.037
CD3+T cell ratio	≥77% vs. <77%	0.802 (0.330–1.952)	0.627	1.737 (0.448–6.742)	0.425
CD4+ T cell ratio	≥41.6% vs. <41.6%	1.024 (0.483–2.171)	0.950	1.073 (0.280–4.117)	0.918
CD8+ T cell ratio	≥29.6% vs. <29.6%	0.672 (0.310–1.458)	0.314	1.087 (0.317–3.736)	0.894
HLADR+ T cell ratio	≥5.8% vs. <5.8%	0.548 (0.275–1.093)	0.088	1.351 (0.394–4.629)	0.632
B cell ratio	≥18.2% vs. <18.2%	1.910 (0.787–4.633)	0.152	1.353 (0.291–6.278)	0.700
NK cell ratio	≥25.6% vs. <25.6%	0.650 (0.227–1.864)	0.423	0.487 (0.062–3.280)	0.493
Edmondson grade	III-IV vs. I-II	1.525 (0.738–3.149)	0.254	3.500 (1.067–11.481)	0.039
Largest tumor size	≥5 cm vs. <5 cm	1.911 (0.901–4.052)	0.091	3.779 (1.128–12.665)	0.031
Microvascular invasion	Yes vs. no	1.144 (0.544–2.406)	0.723	1.926 (0.587–6.324)	0.280
Capsular invasion	Yes vs. no	1.724 (0.863–3.445)	0.123	2.136 (0.645–7.706)	0.214

AFP: alpha-fetoprotein; AST: aspartate aminotransferase; ALT: alanine aminotransferase; GGT: gamma glutamyl transferase.

**Table 7:** Comparisons of clinicopathological characteristics among AFP-positive (≥20 ng/ml) HCC patients with different HLA-DR+ T cell ratio groups.

Variables		Total (*N*)	High HLADR+ T cell ratio (*N*)	Low HLADR+ T cell ratio (*N*)	*χ* ^2^	*P* value
Age	<60 years	62	31	31	0.000	1
	≥60 years	34	17	17		

Gender	Male	71	35	36	0.054	0.816
	Female	25	13	12		

HbsAg	Positive	87	45	42		0.486
	Negative	9	3	6		

Liver cirrhosis	Yes	60	29	31	0.178	0.673
	No	36	19	17		

AST/ALT	<1	58	29	29	0.000	1.000
	≥1	38	19	19		

GGT level	<45 U/L	61	31	30	0.045	0.832
	≥45 U/L	35	17	18		

Child-Pugh grade	A	93	45	48		0.242
	B	3	3	0		

T cell ratio	<77%	75	31	44	10.301	0.001
	≥77%	21	17	4		

CD4+ T cell ratio	<41.6%	60	32	28	0.771	0.399
	≥41.6%	36	16	20		

CD8+ T cell ratio	<29.6%	61	25	36	5.441	0.020
	≥29.6%	35	23	12		

B cell ratio	<18.2%	70	40	30	5.275	0.022
	≥18.2%	26	8	18		

NK cell ratio	<25.6%	85	45	40	2.567	0.109
	≥25.6%	11	3	8		

Largest tumor size	<5 cm	70	32	38	1.899	0.168
	≥5 cm	26	16	10		

Edmondson grade	I-II	47	23	24	0.042	0.838
	III-IV	49	25	24		

Microvascular invasion	Yes	40	17	23	1.543	0.214
	No	56	31	25		

Capsular invasion	Yes	37	19	18	0.044	0.834
	No	59	29	30		

Prophylactic TACE after surgery	Yes	36	18	18	0.000	1.000
	No	60	30	30		

AFP: alpha-fetoprotein; AST: aspartate aminotransferase; ALT: alanine aminotransferase; GGT: gamma glutamyl transferase; TACE: transcatheter arterial chemoembolization.

**Table 8:** Comparisons of clinicopathological characteristics among AFP-negative (<20 ng/ml) HCC patients with different HLA-DR+ T cell ratio groups.

Variables		Total (*N*)	High HLADR+ T cell ratio (*N*)	Low HLADR+ T cell ratio (*N*)	*χ* ^2^	*P* value
Age	<60 years	53	24	29	3.762	0.052
	≥60 years	33	28	15		

Gender	Male	82	44	38	0.058	0.809
	Female	14	8	6		

HbsAg	Positive	77	39	38	1.939	0.164
	Negative	19	13	6		

Liver cirrhosis	Yes	54	32	22	1.289	0.256
	No	42	20	22		

AST/ALT	<1	65	38	27	1.496	0.221
	≥1	31	14	17		

GGT level	<45 U/L	68	39	29	0.953	0.329
	≥45 U/L	28	13	15		

T cell ratio	<77%	75	39	36	0.648	0.421
	≥77%	21	13	8		

CD4+ T cell ratio	<41.6%	64	34	30	0.084	0.772
	≥41.6%	32	18	14		

CD8+ T cell ratio	<29.6%	65	29	36	7.397	0.007
	≥29.6%	31	23	8		

B cell ratio	<18.2%	84	48	36	2.398	0.122
	≥18.2%	12	4	8		

NK cell ratio	<25.6%	83	45	38	0.001	0.980
	≥25.6%	13	7	6		

Largest tumor size	<5 cm	62	39	33	0.000	1.000
	≥5 cm	24	13	11		

Edmondson grade	I-II	69	33	36	3.973	0.046
	III-IV	27	19	8		

Microvascular invasion	Yes	27	15	12	0.029	0.864
	No	69	37	32		

Capsular invasion	Yes	33	19	14	0.235	0.628
	No	63	33	30		

Prophylactic TACE after surgery	Yes	29	13	16	1.460	0.227
	No	67	39	28		

AFP: alpha-fetoprotein; AST: aspartate aminotransferase; ALT: alanine aminotransferase; GGT: gamma glutamyl transferase; TACE: transcatheter arterial chemoembolization.

## Data Availability

The datasets analyzed during the current study are available from the corresponding author upon request.
